# Orthology and paralogy constraints: satisfiability and consistency

**DOI:** 10.1186/1471-2164-15-S6-S12

**Published:** 2014-10-17

**Authors:** Manuel Lafond, Nadia El-Mabrouk

**Affiliations:** 1 Department of Computer Science and Operational Research, University of Montreal, Chemin de la Tour, H3C3J7 Montreal, Canada

**Keywords:** orthology, paralogy, gene tree, species tree, satisfiability, consistency

## Abstract

**Background:**

A variety of methods based on sequence similarity, reconciliation, synteny or functional characteristics, can be used to infer orthology and paralogy relations between genes of a given gene family  G. But is a given set  C of orthology/paralogy constraints possible, i.e., can they simultaneously co-exist in an evolutionary history for  G? While previous studies have focused on full sets of constraints, here we consider the general case where  C does not necessarily involve a constraint for each pair of genes. The problem is subdivided in two parts: (1) Is  C satisfiable, i.e. can we find an event-labeled gene tree G inducing  C? (2) Is there such a G which is *consistent*, i.e., such that all displayed triplet phylogenies are included in a species tree?

**Results:**

Previous results on the *Graph sandwich problem *can be used to answer to (1), and we provide polynomial-time algorithms for satisfiability and consistency with a given species tree. We also describe a new polynomial-time algorithm for the case of consistency with an unknown species tree and full knowledge of pairwise orthology/paralogy relationships, as well as a branch-and-bound algorithm in the case when unknown relations are present. We show that our algorithms can be used in combination with ProteinOrtho, a sequence similarity-based orthology detection tool, to extract a set of robust orthology/paralogy relationships.

## Introduction

Gene families, usually constructed from sequence similarity, group *homologous *genes, i.e., genes sharing a common ancestry: starting from a single gene copy, a history of speciations, duplications and losses is assumed to be at the origin of the observed set of extant genes. Deciphering the *orthology *(divergence following a speciation) and *paralogy *(divergence following a duplication) relations between pairs of genes inside a gene family is important and lies at the heart of many genomics studies. The reconstruction of species trees for example is usually based on the selection and alignment of orthologous gene copies. From a functional point of view, orthologs are believed to be more likely similar in function than paralogs [1]. Orthology/paralogy information is often derived from a reconciliation approach (first introduced by Goodman in 1979 [2]). A gene tree that best reflects the evolution of the sequences is first constructed for the gene family. Assuming a known phylogeny for the set of taxa, the non-agreement between the two trees is then explained by a set of duplication and loss events (other events such as horizontal gene transfer might also be inferred by reconciliation, although we will ignore them here). Reconciliation leads to a labeling of internal nodes of the gene tree as duplication/speciation nodes, yielding a full orthology/paralogy interpretation for each pair of genes (cf. e.g. TreeFam [3,4] used for constructing the *Ensembl Compara *gene trees [5], PHOG [6], MetaPHOrs [7]). This approach assumes that an accurate gene tree can be constructed for the gene family. Although inferring phylogenies is a field with a very long history, due to various limitations constructing good gene trees is still challenging. A variety of other methods have been developed for the purpose of orthology/paralogy detection. A well-known class of algorithms is based on clustering genes according to their sequence similarity (cf. e.g. the COG database [8], OrthoMCL [9], InParanoid [10], Proteinortho [11], orthoDB [12], eggNOG [13]). Recently, we investigated another way of detecting orthology/paralogy based on conserved synteny (conservation in gene order) [14,15]. Other initiatives, such as the Gene Ontology project [16], provide functional annotation that can be used as another source of orthology relations. In contrast to the reconciliation approach, only partial relations can be expected from such tree-free methods.

The orthology/paralogy information suggested by gene tree reconciliation may be contradictory with that suggested by an external source. As gene trees are known to be error-prone, more confidence can be given to such homology information when it is well-supported by various genomic observations. This raises the problem of gene tree editing based on a known set  C of pairwise orthology/paralogy constraints. But prior to any algorithmic consideration, one should be able to state whether the set  C is possible, i.e. whether all constraints can simultaneously co-exist in an evolutionary history of the gene family. In a recent work [14], we showed that a set of orthlogy constraints is always possible and we gave a polynomial-time algorithm for correcting a gene tree in a minimal way according to the Robinson-Foulds distance.

Recent studies have considered the connection of trees and orthology from the angle of reconstructing phylogenies from orthology relations [17-19]: How much information about the gene tree, the species tree and their reconciliation is already contained in the orthology relation between genes? In other words, having a set  C of full pairwise orthology/paralogy relations (each pair of genes is constrained), can one reconstruct the gene and species trees? Similarly to gene tree editing, the first question to be asked is whether the orthology/paralogy constraints can simultaneously co-exist in a history of the gene family. Interestingly, by making the link with symbolic ultrametrics and co-graphs, a simple characterization of *satisfiability *(symbolic ultrametric) for full paralogy/orthology relations is given in [17], where satisfiability relates to the existence of an event-labeled gene tree G (symbolic representation) leading to  C. Notice that satisfiability does not mean the possibility for orthology/paralogy relations to co-exist in a true history, as the triplet phylogenies contained in G are not necessarily *consistent *(included in a species tree). The derivation of a species tree from an event-labeled gene tree is considered in [18]. Finally, the outline of a computational framework for the construction of a least resolved species tree *S *from a set of orthology/paralogy relations, involving the extraction of maximum satisfiable relations and maximum consistent triple set is given in [19].

Here, we consider the general case for  C: in contrast with [17,18], we do not require  C to be full, i.e., to involve a constraint for each pair of genes. We introduce the notations and problems in the following section. We then show how previous results on the *Graph sandwich problem *can be used to solve the problem of satisfiability. The developed algorithm for satisfiability is then adapted to the problem of consistency with a given species tree. In the case of an unknown species tree, we then study the problem of finding a gene tree that is consistent with *some *species tree. Finally we show in the result section how our methodology can be used, in combination with PROTEINORTHO[11], a sequence similarity-based orthology detection tool, to extract a set of robust orthology/paralogy relationships.

## Notations and problem statement

In the rest of this paper, we consider a set Σ of species and a gene family  G where each gene *x *belongs to a species *s*(*x*) of Σ. We generalize the notation *s *to subsets of genes: if *U *⊂  G*, s*(*U*) = {*s*(*x*) : *x *∈ *U*}.

As we only consider rooted trees, we will sometimes omit the word "rooted". Let *T *be a tree. We denote by *r*(*T*) its root and by *L*(*T) *its set of leaves. For any internal node *x *of *T*, we denote by *T_x _*the subtree of *T *rooted at *x*. We say that a node *y *is an *ancestor *of *x *if the two nodes belong to the same path from a leaf to the root of *T*, and *y *is closer to the root. Two nodes *x *and *y *are *unrelated *if *x ≠ y *and none is the ancestor of the other. For a set of leaves *U *⊆ *L*(*T)*, we denote by *lca_T _*(*U*) the *least common ancestral node *of *U *in *T*, i.e. the common ancestral node of the elements of *U *which is the farthest from the root.

Let *L^′ ^*be a subset of *L*(*T*). The *restriction T |_L′ _*of *T *to *L′ *is the tree with leaf set *L′ *obtained from *T*_*lcaT *(*L′*) _ by removing all leaves that are not in *L′*, and all internal nodes of degree 2, except the root. Let *T′ *be a tree such that *L*(*T′*) = *L′ *⊂ *L*(*T)*. We say that *T displays T′ *iff *T |_L′ _*is label-isomorphic to *T′*.

A triplet is a binary tree on a set *L *with *|L| *= 3. For *L *= {*x, y, z*}, we denote by *xy|z *the unique triplet *t *on *L *with root *r*(*t*) for which *lca_t_*(*x, y*) ≠ *r*(*t*) holds. We denote by *tr*(*T*) = *{T|_L _*: $L\in \left(\begin{array}{c} L\left(T\right)\\ 3 \end{array}\right)$ and *T|_L _*is binary*} *the set of all rooted triplets of a tree *T*.

*Evolution of species and genes: *A *species tree S *for Σ is a rooted tree whose leaves are in bijection with Σ, representing the evolutionary relationships between the species: an internal node is an ancestral species at the moment of a speciation event, and its children are the descendants. Although species trees are generally binary, we do not make this assumption here. Genes of  G undergo speciation when the species to which they belong do. Within a species, genes can be duplicated or lost. A *history H *for  G is a tree representing the evolution of the gene family through speciations and duplications: each leaf of *H *is labeled by a gene of  G, and each internal node refers to an ancestral gene at the moment of an event (speciation or duplication). Therefore each internal node of *H *can be labeled as a speciation (*Spec*) or duplication (*Dup*) event.

As *H *is a history "embedded" in the species tree *S *of Σ, it must reflect a speciation history *consistent *with *S*: any speciation node of *H *should reflect a clustering of species in agreement with *S*. To formally define consistency, let first introduce a more general set of labeled trees. We call a *DS-tree *for  G a pair (*G, ℓ*), where G is a tree with *L*(*G*) =  G, and *ℓ *is a function *ℓ *: *V *(*G*) *\ L*(*G*) *→ *{*Dup, Spec*} labeling each internal node of G as a duplication or a speciation node. For simplicity, we often refer to *G *as the *DS*-tree for  G without explicitly stating *ℓ*, and assume the internal nodes of G are labeled *Dup *or *Spec*. For some *X *⊆ *L*(*G*), we implicitly assume that the internal nodes of *G|_X _*share the same label as in *G*.

**Definition 1 ***Let G be a DS-tree for  G and S be a species tree for Σ. We say that G is consistent with S if and only if, for any speciation node x of G and any two children y, z of x, lca_S _(s(L_y_)) and lca_S _(s(L_z_)) are unrelated in S, where L_y _and L_z _are the leaf-sets of G_y _and G_z _respectively.*

Now a history *H *for  G is simply a *DS*-tree for  G consistent with the species tree *S *for Σ. Denote

*tr_S _*(*G*) = {*s*(*x*)*s*(*y*)*|s*(*z*) : *xy|z *∈ *tr*(*G*) is rooted at a speciation and *s*(*x*) ≠ *s*(*y*)}

The triplets of *tr_S _*(*G*) are called *speciation triplets*. The following theorem, which is a reformulation of Theorem 6 in [18], relates consistency to speciation triplets.

**Theorem 1 ***Let G be a DS-tree for  G and S be a species tree for Σ. Then G is consistent with S if and only if S displays all triplets of tr_S _(G).*

From the Fitch [20] terminology, two leaves *x *and *y *of a history are *orthologous *if *lca_H _*(*x, y*) is a speciation node, and *paralogous *otherwise. We extend this terminology to a more general *DS*-tree.

**Definition 2 ***Let G be a DS-tree for  G. Then two genes x, y of G are orthologous with respect to (w.r.t.) G if lca_G_(x, y) is a speciation node, and paralogous w.r.t. G if lca_G_(x, y) is a duplication node.*

Therefore a *DS*-tree *induces *a set of orthology/paralogy relationships between genes.

*Constraint graph: *A *constraint *is simply an unordered pair of genes $\left\{x,\;y\right\}\in \left(\begin{array}{c} G\\ 2 \end{array}\right)$. A set of orthology/paralogy constraints on  G (or simply a constraint set) is a pair C = (*C_O, _C_P_) *of subsets $C_{O},C_{P}\subset \left(\begin{array}{c} G\\ 2 \end{array}\right)$ such that $C_{O}\cap C_{P}=0̸$. *C_O _*represents the *orthology constraints *and *C_P _*the *paralogy constraints*. We say that *C *is a *full constraint set *if $C_{O}\cup C_{P}=\left(\begin{array}{c} G\\ 2 \end{array}\right)$. For example, a history *H *for  G induces a full constraint set.

**Figure 1:**
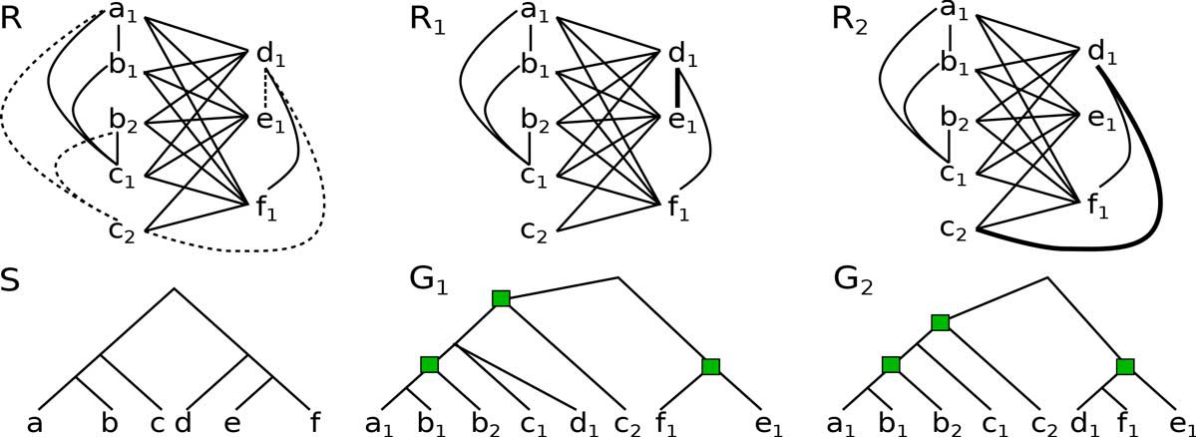
**Constraint graph, satisfiability and consistency**. A constraint graph *R *= (*V, E, U) *with *E *and *U *depicted by solid and dotted edges, along with with two satisfiable realizations *R*_1 _= *R*({*d*_1_*e*_1_}) and *R*_2 _= *R*({*d*_1_*c*_2_}). The gene names correspond to their respective species in the species tree *S*. The tree *G*_1 _is a *DS*-tree that satisfies *R*_1 _while *G*_2 _is a *DS*-tree that satisfies *R*_2_. Duplication nodes in *G*_1 _and *G*_2 _are indicated by a green square. *G*_1 _is not consistent with *S *because for instance, *G*_1 _has the speciation triplet *s*(*a*_1_)*s*(*d*_1_)*|s*(*e*_1_) = *ad|e *while *S *has the *de|a *triplet. The tree *G*_2 _is consistent with *S*.

We represent a constraint set *C *= (*C_O, _C_P_)*by an edge-bicoloured undirected graph *R *= (*V, E, U)*, called a *constraint graph*, with vertex set *V *=  G, and two edge sets *E *= *C_O _*and $U=\left(\begin{array}{c} G\\ 2 \end{array}\right)\backslash \left(C_{O}\cup C_{P}\right)$. Said differently, two genes are linked by an edge of *E *if they are constrained by orthology, unlinked if they are constrained by paralogy, and linked by a "special" edge of *U *if the relation between the two genes is unknown. We refer to the edges of *E *as the *orthology edges*, to those in *U *as the *unknown edges *and we refer to the unlinked pairs of genes as the *paralogy non-edges*. An example of a partial constraint graph is given in Figure 1.

If *C *is a full constraint set then U=0̸. *R *is called a *full constraint graph *in this case. The *complement *of *R *is the graph $\bar{R}$ obtained by the alternative choice of linking paralogs instead of orthologs. Formally, $\bar{R}=\left(V,Ē\backslash U,U\right)$, where  Ē is the complement of *E *defined by *e *∈  Ē iff *e *∉ *E*. Notice that *R *and $\bar{R}$ share the same set *U *of unknown edges. We denote by *R*[*X*] the graph *R induced *by *X *⊆ *V*, i.e. *R*[*X*] = (*X, E*(*X*)*, U *(*X*)) where *E*(*X*) (resp. *U *(*X*)) are the edges of *E *(resp. *U) *having both endpoints in *X*. Note that if U=0̸, *R*[*X*] corresponds to the usual definition of the graph induced by *X*.

*Satisfiability and consistency *Given a constraint set *C *(or similarly a constraint graph *R*), is *C possible*, i.e. can we find a history for  G inducing the orthology and paralogy constraints of *C*? As an orthology constraint for two genes belonging to the same genome cannot be induced by a history for  G, we assume in the rest of this paper that the set ${\tilde{C}}_{P}=\left\{\left\{x,y\right\}\in \left(\begin{array}{c} G\\ 2 \end{array}\right):s\left(x\right)=s\left(y\right)\right\}$ is included in *C_P_*. A *trivial set of paralogy constraints *is a set *C_P _*restricted to ${\tilde{C}}_{P}$.

The question whether *C *is possible is in two parts: (1) is *C satisfiable*, i.e. can we find a *DS*-tree *G *inducing  C and (2) is there such a *G *which is consistent with a species tree? Formal definitions follow.

**Definition 3 ***Let R = (V, E, U) be a constraint graph and G be a DS-tree with L(G) = V. We say that G satisfies R if for two genes x, y ∈ L(G), if xy ∈ E then they are orthologous w.r.t. G, and if xy ∈ E \ U then they are paralogous w.r.t. G. We say that R is satisfiable if there exist a DS-tree G that satisfies R.*

If U=0̸, then *R *being satisfiable means that we can make a choice for the unknown edges as orthology edges and paralogy non-edges to obtain a full constraint graph that is satisfiable. For *F *⊂ *U*, the *realization *of *R *by *F *corresponds to choosing *F *as orthology edges, and *U \ F *as paralogy non-edges, leading to the full constraint graph R(F)=(V, E∪F,0̸). We call R(0̸) and *R*(*U*) the *empty *and *full *realizations, respectively.

As a history is a *DS*-tree, a set of constraints that is not satisfiable is clearly not possible, i.e. there is no history that depicts the orthology/paralogy relationships given by the constraints. Moreover, satisfiability is not sufficient to ensure the possibility of such an history, as a *DS*-tree is not always consistent with a species tree. Figure 1 shows an example of a constraint graph *R *along with two satisfying realizations *R*_1 _and *R*_2_. However, *R*_1 _cannot be made consistent with a given species *S *whereas *R*_2 _can.

**Definition 4 ***Let R be a constraint graph for  G. We say that R is consistent with a species tree S if and only if there is a realization of R which is satisfiable by a DS-tree G which is consistent with S. More generally, we say that R is consistent if and only if there is a species tree S such that R is consistent with S.*

The three following sections are respectively dedicated to the three following questions: (1) Given a constraint graph *R *= (*V, E, U*), is *R *satisfiable? (2) Given a satisfiable constraint graph *R *= (*V, E, U*), and a species tree *S*, is *R *consistent with *S*? (3) Given a satisfiable constraint graph *R *= (*V, E, U*), is *R *consistent, i.e. can we find a species tree *S *such that *R *is consistent with *S*?

## Satisfiability of a constraint graph

The problem of constraint graph satisfiability has been addressed in [17] in the restricted case of a full set of constraints. The following theorem is a reformulation of one of the main results of this paper.

**Theorem 2 (**[17]**) ***A full constraint graph R is satisfiable if and only if R is P_4_- free (or equivalently, iff $\bar{R}$ is P_4_-free since P_4 _is self-complementary), meaning that no four vertices of R induce a path of length 4.*

Consider now the general case of a constraint graph *R *= (*V, E, U) *with U=0̸. Then the problem is to find a realization *R*(*F*) that is itself satisfiable, i.e. *P*_4_- free. It turns out that this problem is a reformulation of the well-known *Graph sandwich problem *for *P*_4_-free graphs. It can be stated as follows : given two graphs *G*_1 _= (*V, E*_1_) and *G*_2 _= (*V, E*_2_), with *E*_1 _⊆ *E*_2_, does there exist a *P*_4_-free graph *G *= (*V, E*) such that *E*_1 _⊆ *E *⊆ *E*_2_. That is, G must contain every edge of *G*_1 _and every non-edge of *G*_2_. It is then clear that this is equivalent to finding a *P*_4_-free realization of *R *= (*V, E*_1_*, U *= *E*_2 _*\ E*_1_). A *O*(*|V |*^3^) algorithm was proposed in [21] to solve this problem. In this section, we restate under our formalism some of the useful results of this paper, and give a modified version of the proposed algorithm that outputs a *DS*-tree that satisfies *R *whenever there is one. We will make use of the following well-known characterization of *P*_4_-free graphs.

**Lemma 1 ***A graph Γ is P_4_-free if and only if, for any subset X of vertices of Γ with |X| ≥ 2, either Γ[X] or $\bar{\Gamma \left[X\right]}$ is disconnected.*

The next lemmata establish an important *heritability *property on satisfiable graphs: every restriction *R*[*X*] of *R *must be satisfiable for *R *to be satisfiable.

**Lemma 2 ***Let G be a DS-tree that satisfies a realization R(F), for some F ⊆ U. Let X ⊆ V and let F_X _= {ab ∈ F : a, b ∈ X}. Then G|_X _is a realization of R[X](F_X_).*

*Proof: *Let *a, b ∈ X*. First observe that *a *and *b *have the same orthology/paralogy relationship in *R*(*F) *and *R*[*X*](*F_X_)*. Let *c *= *lca_G_*(*a, b*). Now, *c *is also an internal node of *G|_X, _*and moreover *c *= *lca_G|X_*(*a, b*). As *c *has the same *Dup *or *Spec *label as in *G, c *correctly displays the relationship between *a *and *b*. Thus *G|_X _*satisfies every relationship in *R*[*X*](*F_X_*).

The heritability property is then immediate.

**Lemma 3 **[21]*A constraint graph R = (V, E, U) is satisfiable if and only if for any X ⊆ V, R[X] is satisfiable.*

**Theorem 3 **[21]*A constraint graph R is satisfiable if and only if at least one of the two following holds :*

1 R(0̸) is disconnected, and all of its connected components are satisfiable;

2 $\bar{R\left(U\right)}$ is disconnected, and all of its connected components are satisfiable.

*Proof: ⇐ *For 1.: Suppose R(0̸) is disconnected. Let {*R*_1_*,..., R_k_*} be the connected components of R(0̸) with *k >*1, all being satisfiable. As each *R_i _*is satisfiable, there is a *DS*-tree *G_i _*that satisfies a realization *R_i_*(*F_i_*) of *R_i_*. Let *F *= ∪_1*≤i≤k *_*F_i_*. Then the realization *R*(*F*) of *R *is a full constraint graph with *k *full constraint components *R_i_*(*F_i_*) in which no two components share an edge. In other words, there is a paralogy non-edge between each pair of genes from two different components. Therefore, joining the roots of *G*_1_*,..., G_k _*under a common duplication node yields a *DS*-tree for *R*(*F*), which shows that *R *is satisfiable. The proof when 2. holds is the same, except that we root G at a speciation node since the components of $\bar{R\left(U\right)}$ are pairwise-complete in *R*(*U*).

*⇒ *Suppose that both conditions do not hold. If R(0̸) or $\bar{R\left(U\right)}$ has a component that is not satisfiable, then by Lemma 3, *R *is not satisfiable. So instead suppose that each of R(0̸) and $\bar{R\left(U\right)}$ has a single connected component. Let *F *⊆ *U*. The realization *R*(*F*) of *R *must be connected as R(0̸) is already connected and E(R(0̸))⊆E(R(F)). $\bar{R\left(F\right)}$ must also be connected, as choosing all edges of *U *leaves $\bar{R\left(U\right)}$ connected and $E\bar{R\left(U\right)}\subseteq E\bar{\left(R\left(E\right)\right)}$. Since both *R*(*F*) and $\bar{R\left(F\right)}$ are connected, by Lemma 1 *R*(*F*) is not *P*_4_-free, and thus not satisfiable by Theorem 2. As this is true for any realization *R*(*F*) of *R*, i.e. for any *F *⊆ *U*, it follows that *R *is not satisfiable.

Theorem 3 suggests the recursive algorithm BuildDSTree that begins by finding out if one of R(0̸) or $\bar{R\left(U\right)}$ is disconnected. If so, it creates a node of the appropriate type with children being the identified components, and repeats the process on each such component.

Algorithm BuildDSTree (*R *= (*V, E, U*), *v*)

where *R *is a (possibly induced) constraint graph and *v *is the current node of *G *we are creating

IF *|V | *= 1; RETURN;

R(0̸)=(V,E,0̸)

Find the connected components *CC *of R(0̸) through a depth-first search

IF |CC| > 1;

type ← Dup

ELSE

$\bar{R\left(U\right)}=\left(V,\;Ē,0̸\right)$

type ← Spec

Find the connected components *CC *of $\bar{R\left(U\right)}$ through a depth-first search IF *|CC| *= 1; output "Unsatisfiable", and halt the recursion

END IF

v.type ← type

FOR C ∈ CC;

Add child node *v_C_*to *v*

BuildDST ree(R[C], vC)

END FOR

RETURN

If *n *is the number of genes in *G*, the algorithm creates a *DS*-tree *G *with at most *n − *1 internal nodes (or stops before if *R *is unsatisfiable). For each such internal node *v*, the time taken to go through the algorithm is dominated by (at most) two depth-first searches that are performed on *L*(*G_v_)*, and the rest of the work is handled by children nodes. So the time taken to handle *v *is bounded by the number of edges/non-edges in *R*[*L*(*G_v_)*], which is *O*(*|L*(*G_v_)|*^2^) ⊆ *O*(*n*^2^). Therefore the time-complexity of BuildDSTree is in *O*(*n*^3^).

## Consistency with a given species tree

Let *R *= (*V, E, U) *be a constraint graph for  G and *S *be a species tree for Σ. We want to know whether the orthology/paralogy constraints represented by *R *can be induced by a history for  G consistent with *S*. More precisely, is there a realization *R*(*F) *of *R *that is satisfiable and such that the DS-tree satisfying *R*(*F) *is consistent with *S*? If *R *is not satisfiable, then the answer is clearly no. Therefore hereafter we assume that *R *is satisfiable. We first show that the problem at hand still has the heritability property.

**Lemma 4 ***R is consistent with S if and only if for any X ⊆ V, R[X] is consistent with S.*

*Proof: *The '⇐' part is trivial since we can choose *X *= *V *to show that *R *is consistent with *S*. Conversely, assume *R *is consistent with *S*. Let G be a *DS*-tree for some realization of *R *such that G is consistent with *S*, and let *X *⊆ *V*. By Lemma 2, *G|_X _*is a *DS*-tree for *R*[*X*]. Let *ab|c *∈ *tr_S _*(*G|_X_)*. Since going from *G|_X _*to *G *only involves adding subtrees on branches of *G|_X, _*it follows that *ab|c *∈ *tr_S _*(*G*). Therefore, *tr_S _*(*G|_X_) *⊆ *tr_S _*(*G*). Now, since *S *displays *tr_S _*(*G*), *G|_X _*is a realization of *R*[*X*] that is consistent with *S*.

We need to introduce one last notation before stating the main theorem for characterizing consistency of a constraint graph *R *with a species tree *S*. Let *R*(*F*) be a realization of *R*, and let *CC *= {*R*_1_*,..., R_k_*} be the connected components of $\bar{R\left(F\right)}$. Notice that the components of *CC *are pairwise complete in *R*(*F*). A *speciation partition P *= {*P*_1_*,..., P_|P|_*} is a non-trivial partition of *CC *(i.e. *|P | >*1) such that *lca_S _*(*s*(*P_i_*)) is unrelated to *lca_S _*(*s*(*P_j_*)) whenever *i *≠ *j*.

**Theorem 4 ***R is consistent with S if and only if at least one of the following conditions holds:*

1 R(0̸) is disconnected and each connected component is consistent with S;

2 $\bar{R\left(U\right)}$ is disconnected, its components admit a speciation partition and each component in this partition is consistent with S.

*Proof: *⇐ For 1., Let {*R*_1_*,..., R_k_*} be the connected components of R(0̸), each *R_i _*having a *DS*-tree *G_i _*consistent with *S*. We can then join the roots of *G*_1_*,..., G_k _*under a common duplication parent. This yields a *DS*-tree G that satisfies *R *as each pair of components of R(0̸) are related by paralogy. Furthermore, all rooted triplets of *G *that were not in any *G_i _*are rooted at *r*(*G*), a *Dup *node. Therefore, *tr_S _*(*G*) = *∪*_1*≤i≤k *_*tr_S _*(*G_i_*), which *S *displays.

⇐ 2.: Let *P *= {*P*_1_*,..., P_k_*} be a non-trivial speciation partition of the connected components of $\bar{R\left(U\right)}$. By assumption every *P_i _*∈ *P *has a *DS*-tree *G_i _*that is consistent with *S*, implying that *S *displays *∪*_1≤*i*≤*k *_*tr_S _*(*G_i_*). Here all elements of *P *are components of *R *that are pairwise-complete, and we obtain a *DS*-tree G for *R *by joining *G*_1_*,..., G_k _*under a common speciation parent. Let *T *= *tr_S _*(*G*) *\ ∪*_1≤*i*≤*k *_*tr_S _*(*G_i_*). Every triplet of *T *is rooted at *r*(*G*). Thus if three genes *a, b, c *of *L*(*G*) form a speciation triplet *s*(*a*)*s*(*b*)*|s*(*c*) *∈ T*, then *a *and *b *are in some part *P_i _*while *c *is in another part *P_j_*. But by the definition of speciation partitions, *lca_S _*(*s*(*P_i_*)) is unrelated to *lca_S _*(*s(P_j_*)), implying that *s*(*a*)*s*(*b*)*|s*(*c*) ∈ *tr*(*S*). It follows that *S *displays *T*.

⇒ : suppose both conditions are not met, but that *R *is consistent with *S*. If R(0̸) is disconnected but has an inconsistent component, then *R *is inconsistent by Lemma 4. So we assume R(0̸) is connected. If $\bar{R\left(U\right)}$ is also connected, then we saw in Theorem 3 that *R *is not even satisfiable. If $\bar{R\left(U\right)}$ is disconnected and admits a speciation partition, but a member of this partition is not consistent, then again by Lemma 4, *R *is not consistent. So we assume that R(0̸) is connected, and $\bar{R\left(U\right)}$ is disconnected but admits no speciation partition. Let *G *be a *DS*-tree for *R *consistent with *S*. Suppose *r*(*G*) is a duplication node and let *r*_1_*, r*_2 _be two children of *r*(*G*). We have that every gene in *L*(*G*_*r*_1__) is paralogous with every gene in *L*(*G*_*r*_2__) and vice-versa. This implies that *L*(*G*_*r*_1__) and *L*(G_*r*_2__) are two components of R(0̸) that share no edge, a contradiction since we assume R(0̸) is connected. So *r*(*G*) is a speciation node. Let *r*_1_*,..., r_k _*be the children of *r*(*G*). The sets *L*(*G*_*r*_1__),..., *L*(*G*_*r_k_*_) form a partition *P *of the connected components of $\bar{R\left(U\right)}$. Since *S *displays *tr_S _*(*G*), it follows that for two distinct *P_i_, P_j _*∈ *P, lca_S _*(*s*(*P_i_*)) and *lca_S _*(*s*(*P_j_)*) are unrelated.

Hence *P *is a speciation partition, a contradiction.

This theorem suggests a small modification to algorithm BuildDSTree. Connected components of R(0̸) are handled in the same manner, but in the case of a disconnected $\bar{R\left(U\right)}$, we need to find a speciation partition *P *after having found its connected components *CC*. To accomplish this, it suffices to observe that some *C*_1_*, C*_2 _∈ *CC *must be in the same part of *P *when *lca_S _*(*s*(*C*_1_)) is on the path from *lca_S _*(*s*(*C*_2_)) to the root of *S *(or vice-versa). Thus for each *P_i _*∈ *P*, we can find the member of *C *∈ *P_i _*that has *lca_S _*(*s*(*C*)) the closest to the root of *S*, then any other component *C′ *having *lca_S _*(*s*(*C′*)) in the subtree rooted at *lca_S _*(*s*(*C*)) will be in *P_i_*.

FINDSPECIATIONPARTITION uses that fact to find *P *through a pre-order traversal of *S*.

Algorithm FindSpeciationPartition(*CC, s, P, Pi*)

where *CC *is the set of components to partition, *s *∈ *V *(*S*) is the current node of *S *in the pre-order traversal, *P *is the partition of *CC*, and *Pi *is the current part of *P *we are adding components to

FOR *C *∈ *CC *such that *lca_S_*(*C*) = *s*;

IF *P_i _*is not set; let *P_i _*be a new empty set and add *P_i _*to *P*

Add *C *to *P_i_*

END FOR

FOR s′ ∈ children(s);

FindSpeciationPartition(*CC, s′, P, P_i_*)

END FOR

Assuming constant time for each *lca *lookup, we can precompute *lca_S _*(*s*(*C*)) in time *|C| *for each *C *∈ *CC*. If *CC *has a total of *k *nodes, by mapping each *s *∈ *S *to the list of *C *∈ *CC *with *lca_S _*(*s*(*C*)) = *s*, the whole algorithm takes time *O*(*k *+ *|S|*). We need up to *n − *1 calls of BuildDSTree. We argued that one call on a node *v *of *G *in BUILDDSTREE takes time *O*(*|L*(*G_v_)|*^2^), so adding this step makes it *O*(*|L*(*G_v_)|*^2 ^+ *|S| *+ *k*). Noting that *k *= *|L*(*G_v_)|*, and assuming that *|L*(*G_v_)| ≥ |S|*, this modified algorithm still runs in time *O*(*n*^3^), where n=|G|.

## Consistency of a satisfiable constraint graph

Now let *R *= (*V, E, U) *be a constraint graph for  G and suppose the species tree for Σ is unknown. The question is to know whether the graph *R *is consistent, and if so to output a species tree *S *such that *R *is consistent with *S*. As above, we assume that *R *is satisfiable. Note that unlike the two previous problems, we cannot treat each connected component of R(0̸) or $\bar{R\left(U\right)}$ independently, as two (or more) components might give gene histories consistent by themselves but not together.

We hereafer begin with two classes of constraint graphs for which consistency always holds.

*Orthology constraints only: *Suppose R=(V=G,E,U) represents a constraint set restricted to orthology constraints, i.e. *C *= (*C_O, _C_P_)*, where $C_{P}={\overset{\sim }{C}}_{P}$ is the trivial set of paralogy constraints. For each *s_i _*∈ Σ, let $L_{s_{i}}=\left\{x\in V:s\left(x\right)=s_{i}\right\}$ and let $F_{s_{i}}$ be the star-tree joining all the leaves of $L_{s_{i}}$ under a single duplication node. Now let *G *be the star-tree joining all the $F_{s_{i}}$ trees, for all *s_i _*∈ Σ, under a single speciation node. Then *G *satisfies the full realization *R*(*U*) of *R *and it is consistent with the star-tree for Σ. Therefore any set of orthology constraints is consistent.

*Paralogy constraints only: *Suppose R=(V=G,E,U) represents a constraint set restricted to paralogy constraints, i.e. $C=\left(C_{O}=0̸,\;C_{P}\right)$. If *G *is the star-tree joining all the genes of  G under a single duplication node, then *G *satisfies the empty realization R(0̸) of *R *and it is consistent with the star-tree for Σ. Therefore any set of paralogy constraints is consistent.

Consider now a full constraint graph *R*. The results in [17,18] suggest a polynomial-time algorithm for solving the consistency problem that consists in building a *DS*-tree *G *satisfying *R*, extracting all speciation triplets of *G *and checking their consistency with a species tree. Here we propose an alternative polynomial time algorithm for the same problem, avoiding the first step of a *DS*-tree construction. We first introduce the following subset *P*_3_(*R*) of triplets of $\left(\begin{array}{c} V\\ 3 \end{array}\right)$ inducing a path of size 3 in *R*:

$$P_{3}\left(R\right)=\left\{s\left(x\right)s\left(y)\right|s\left(z\right)\;:\left\{x,y,z\right\}\in \left(\begin{array}{c} V\\ 3 \end{array}\right),zx,zy\in E\;\text{and}\;xy\notin E\cup U\;\text{and}\;s(x)\notin s\left(y\right)\right\}$$

Notice that *s*(*x*)*s*(*y*)*|s*(*z*) ∈ *P*_3_(*R*) implies that any *DS*-tree *G *satisfying *R *has *s*(*x*)*s*(*y*)*|s*(*z*) ∈ *tr_S _*(*G*). Indeed, since *xy *∉ *E *∪ *U, lca_G_*(*x, y*) is a duplication node. And since both *x *and *y *are related to *z *by speciation, *lca_G_*(*x, z*) = *lca_G_*(*y, z*) and *xy|z *must be a speciation triplet of *G*.

For example, consider the vertices *b*_1_*, c*_2_*, e*_1 _of the *R *graph in Figure 1, which form a path of length 3 with *e*_1 _in the center. In the *DS*-tree *G*_1_, $lca_{G_{1}}\left(b_{1},\;c_{2}\right)$ is a duplication, and $lca_{G_{1}}\left(b_{1},\;c_{2}\right)$ ({*b*_1_*, c*_2_*, e*_1_}) is a speciation. Restricting *G*_1 _to the three vertices yields the triplet *b*_1_*c*_2_*|e*_1 _rooted at a speciation, and therefore, *s*(*b*_1_)*s*(*c*_2_)*|s*(*e*_1_) ∈ *tr_S _*(*G*_1_).

The same holds for the *s*(*c*_1_)*s*(*c*_2_)*|s*(*e*_1_) triplet implied by the *P*_3 _induced by *c*_1_*, c*_2_*, e*_1_. Notice however that in both *DS*-trees, *s*(*b*_1_)*s*(*c*_1_)*|s*(*e*_1_) is a speciation triplet, though *b*_1_*, c*_1_*, e*_1 _do not induce a *P*_3_. We show that this kind of speciation triplet is implied by the other two aforementioned *P*_3_, and that the *P*_3 _subgraphs actually imply every mandatory speciation triplet.

**Theorem 5 ***Let R=(V,E,U=0̸) be a satisfiable full constraint graph. Then R is consistent if and only if there exists a species tree S displaying all the triplets of P_3_(R).*

*Proof: *⇒ : since *s*(*x*)*s*(*y*)*|s*(*z*) ∈ *P*_3_(*R*) implies that *s*(*x*)*s*(*y*)*|s*(*z*) ∈ *tr_S _*(*G*), it follows that any species tree *S *consistent with *R *must display every triplet of *P*_3_(*R*).

⇐ : we first obtain a least-resolved *DS*-tree *G *for *R *in terms of speciation. Let *G′ *be a consistent *DS*-tree satisfying *R*, and let *S *be a species tree displaying *P*_3_(*R*). If *G′ *has any speciation node *v *that has a speciation child *w*, we obtain *G″ *by contracting *v *and *w *(delete *w *and give its children to *v*). Since *v *and *w *are both speciations, this operation does not change the label of *lca_G_*(*x, y*) for any two leaves *x *and *y *and *G″ *still satisfies *R*. Moreover, *tr_S _*(*G″*) ⊂ *tr_S _*(*G′*), so there is no risk of breaking consistency. We obtain a *DS*-tree G by repeating this operation until we cannot find such a *v *and *w*.

Let *xy|z *be a triplet of *G *rooted at a speciation node. We have that *lca_G_*(*z, x*) = *lca_G_*(*z, y*) is a speciation, and that *zx, zy *∈ *E*. If *lca_G_*(*x, y*) is a duplication node, then *xy *∉ *E*. So {*x, y, z*} induces a *P*_3 _in *R*, and *S *displays *s*(*x*)*s*(*y*)*|s*(*z*). Suppose instead that *lca_G_*(*x, y*) is a speciation node. Because *G *is a least resolved *DS*-tree, there must be a duplication node *u *on the path between *lca_G_*(*x, y*) and *lca_G_*(*x, z*). This implies there is a leaf *d *in *G_u _*such that *x *and *y *are related to *d *by duplication, but with *d *and *z *related by speciation. In *R*, we then have *zd *∈ *E*, and *xd, yd *∉ *E*. Thus both {*x, d, z*} and {*y, d, z*} induce a *P*_3 _in *R *with *z *being the middle vertex, and *s*(*x*)*s*(*d*)*|s*(*z*)*, s*(*y*)*s*(*d*)*|s*(*z*) ∈ *P*_3_(*R*) are both displayed by *S*. This is only possible if there is a node in *S *that has all of *s*(*x*)*, s*(*y*)*, s*(*d*) in one child subtree and *s*(*z*) in another. Therefore, *S *must display *s*(*x*)*s*(*y*)*|s*(*z*). Having taken care of both types of speciation triplets, we deduce that displaying *P*_3_(*R*) is sufficient to display *tr_S _*(*G*).

Therefore the consistency problem for a satisfiable full constraint graph reduces to the problem of verifying whether the set *P*_3_(*R*) of triples can be displayed in a species tree for Σ. This is in fact a well know problem with a solution presented in [22]: given a triplet set  R, there is a polynomial-time algorithm, called BUILD [23], that, when applied to  R either outputs a species tree that displays  R or recognizes that  R is inconsistent. Therefore, in the case of a full constraint graph, the consistency problem is resolved in polynomial time by first constructing the set *P*_3_(*R*), and then applying the BUILD algorithm.

Consider now the general case of a constraint graph *R *= (*V, E, U) *with U≠0̸. The branch-and-bound algorithm CHECKCONS iterates over the edges of *U*, tries to make them edges and non-edges but stops as soon as one decision creates a set of *P*_4_ with no unknowns or a set of *P*_3 _that is inconsistent. Since at worst, every possibility is tested, it follows that this algorithm is exact, though exponential in the worst case.

ALGORITHM CHECKCONS (*R *= (*V, E, U*))

Obtain a species tree *S *by running BUILD on *P*_3 _(*R*)

IF *S *is not set (i.e. BUILD failed), RETURN FALSE

IF *R *is not satisfiable, RETURN FALSE

IF U=0̸, return (*R, S*)

Let e ∈ U and let R_e_ = (V, E ∪ {e}, U \ {e})

(R′, S) ← CHECKCON S(R_e_)

IF (*R′, S*) is set (i.e. CHECKCONS succeeded), RETURN (*R′, S*) Otherwise let $R_{ē}=\left(V,E,U\backslash \left\{e\right\}\right)$

(R′, S) ← CHECKCONS$\left(R_{ē}\right)$

IF (*R′, S*) is set (i.e. CHECKCONS succeeded), RETURN (*R′, S*)

Otherwise RETURN FALSE

Possible improvements of this algorthm include removing as many edges from *U *as possible, and choosing an ordering of the edges that may speed up the branch-and- bound process. For instance, it may be worthwhile to first identify every induced *P*_4 _of R(0̸). The *P*_4 _subgraphs that admit only one possibility for removal, i.e. the *P*_4 _can only be removed by making a unique edge *e *∈ *U *an orthology edge, can be corrected before entering the algorithm. Note that the same applies for the edges of *U *that must be edges of $\bar{R\left(U\right)}$. We may then prioritize the handling of the other *P*_4 _by considering the edges that resolve them first. Similarly, it would also be possible to identify edges of *U *that are mandatory in *E *by finding the *P*_3 _subgraphs of R(0̸) that are not in *P*_3_(*R*), but that disagree with a triplet of *P*_3_(*R*). For instance, R(0̸) might have a *P*_3 _with edges *xz, zy*, but this *P*_3 _is not in *P*_3_(*R*) because *xy *∈ *U*. If say *s*(*y*)*s*(*z*)*|s*(*x*) is in *P*_3_(*R*), then *xy *is forced in *E *as otherwise the contradictory *s*(*x*)*s*(*y*)*|s*(*z*) triplet would be present.

## Experiments

We show how the developed algorithms for checking satisfiability and consistency can be used, in combination with an orthology detection tool such as ProteinOrtho [11], to infer a robust set of orthology and paralogy constraints. Given a set of protein sequences, Proteinortho infers homologous gene families as well as orthology relationships within these families, based on various similarity scores. Proteinortho does not infer paralogy relationships. However, if we choose a set of parameters leading to a loose characterization of orthologs, then we can assume that unpredicted constraints should represent paralogy. Different combinations of parameters therefore lead to different constraint sets that can be analyzed for sat-isfiability and consistency.

ProteinOrtho has been run on 265 gene families of vertebrates, each representing the leaf-set of an *Ensembl *[5] gene tree. Trees were chosen randomly among the *Ensembl *gene trees containing at least 20 leaves. For each family, five different parameter settings, numbered from *−*2 to +2, were tested, 0 representing the default parameter choice of ProteinOrtho, and the smaller the number, the looser is the induced characterization of orthology. For each parameter setting *i*, we define the full constraint graph *R^i ^*where all gene pairs not predicted as orthologs are interpreted as paralogs. Typically, a graph *R^− ^*for a negative number (*−*1 or *−*2) contains more orthology (and thus less paralogy) constraints than *R*^0^, while the converse is true for a graph *R*^+^. Combining two constraint graphs *R^− ^*and *R*^+ ^consists in keeping only orthology and paralogy edges that are common to both, and completing the graph with unknown edges.

Table 1 summarizes the results on satisfiability and consistency with the *Ensembl *species tree *S*, obtained for each gene family and each parameter setting or combination. Among the 265 gene families, only 112 (42%) produced at least one satisfiable full constraint graph and only 44 (15%) produced such a graph which is also consistent with the *Ensembl *species tree. However, combining loose and strict parameter settings lead to much better results with at least 95% satisfiability and 56% consistency with *S*. The partial orthology/paralogy constraints obtained from combinations correspond to about half of the constraints of a full graph, as illustrated by the last column of the table.

**Table 1:** The results over 265 gene families from Ensembl.

	# satisfiable families	# consistent families	% constraints when consistent
−2	82 (30.9%)	30 (11.3%)	
−1	44 (16.6%)	13 (4.91%)	
0	26 (9.81%)	9 (3.40%)	
+1	48 (18.1%)	14 (5.28%)	
+2	55 (20.8%)	18 (6.79%)	
−2/+2	260 (98.1%)	172 (64.9%)	42.0%
−2/+1	258 (97.4%)	172 (64.9%)	44.8%
−1/+1	254 (95.8%)	149 (56.2%)	50.6%
−1/+2	255 (95.9%)	157 (59.2%)	47.5%

The first five rows correspond to the full constraint sets obtained from PROTEINORTHO for the five classes of parameters. The last four rows correspond to the partial constraint sets obtained after combining two graphs over two types of parameters. The first column is the number of families for which the settings of PROTEINORTHO yielded a satisfiable graph, the second that number consistent with the Ensembl species tree. The last column shows the percentage of constraints that were not unknown when a consistent solution was found (which is 100% for the first five rows).

In order to get a rough idea of the accuracy of the obtained partial orthology/paralogy predictions for each gene family  G, we compared them with those resulting from the labeling of the *Ensembl *gene tree nodes as duplication and speciation nodes. An *orthology disagreement *refers to orthology predictions on the four combined graphs depicted in Table 1, that are rather inferred as paralogs from the *Ensembl *gene tree labeling. A *paralogy disagreement *refers to the reverse situation. Overall, the orthology disagreement percentage is between 15.1% and 15.9% depending on the two classes of parameters combined. For paralogy disagreement, it varies between 11% and 17%, depending on the 2 parameters combined (−2/+1 and −2/+2 were around 11.2% while −1/+1 and −1/+2 were around 17.4%).

Notice that *Ensembl *annotates many duplication nodes as "dubious". If we ignore orthology disagreements caused by a dubious duplication node, the orthology disagreement percentage drops to an average of 5.0%, strengthening the doubts on those duplication nodes.

## Conclusion

In this work we have developed methods to assess the plausibility of a partial set of orthology and paralogy relationships between pairs of homologous genes. In particular, we showed how extending algorithms for the *Graph sandwich problem *can solve, in cubic time, the problems of satisfiability and consistency with a given species tree. In case of an unknown species tree, the complexity of the problem of verifying consistency with *some *species tree remains open. We have elaborated on the *P*_3 _property of the constraint graph, which leads to an exponential branch-and- bound algorithm. It remains possible that this property could be used to create a more efficient method. While previous work consisted in verifying whether a full set of relationships was satisfiable or consistent, admitting uncertainty within these relationships makes it possible to bring the theory from [18] into practice, as current orthology (or paralogy) inference methods based on sequences cannot guarantee 100% accuracy in their predictions. We show how a confidence set of such predictions can be inferred using our methods and Proteinortho. A promising direction is to use such robust predictions to correct gene trees in case of disagreement.

## Availability

Software is available at http://www-ens.iro.umontreal.ca/~lafonman/software.php.

## Competing interests

The authors declare that they have no competing interests.

## Authors' contributions

ML, NE devised the proofs and algorithms and wrote the paper. ML implemented the software.

## Declarations

Publication charges for this work was funded by the Natural Sciences and Engineering Research Council of Canada (NSERC) Discovery Grant and Fonds de Recherche Nature et Technologies of Quebec (FQRNT) B2 Grant.

This article has been published as part of *BMC Genomics *Volume 15 Supplement 6, 2014: Proceedings of the Twelfth Annual Research in Computational Molecular Biology (RECOMB) Satellite Workshop on Comparative Genomics. The full contents of the supplement are available online at http://www.biomedcentral.com/bmcgenomics/supplements/15/S6.
